# Tauroursodeoxycholic Acid Decreases Keloid Formation by Reducing Endoplasmic Reticulum Stress as Implicated in the Pathogenesis of Keloid

**DOI:** 10.3390/ijms221910765

**Published:** 2021-10-05

**Authors:** Sunje Kim, Seong Eun Lee, Shinae Yi, Sangmi Jun, Yoon-Sun Yi, Harsha Nagar, Cuk-Seong Kim, Chungmin Shin, Min-Kyung Yeo, Yea Eun Kang, Sang-Ha Oh

**Affiliations:** 1 Department of Plastic and Reconstructive Surgery, College of Medicine, Chungnam National University, Daejeon 35015, Korea; ksj9243@gmail.com (S.K.); piglet7474@gmail.com (C.S.); 2 Research Institute for Medicinal Sciences, College of Medicine, Chungnam National University, Daejeon 35015, Korea; seongeun316@cnu.ac.kr (S.E.L.); shinae1069@gmail.com (S.Y.); 3 Center for Research Equipment, Korea Basic Science Institute, Daejeon 34133, Korea; smjun@kbsi.re.kr (S.J.); yys02@kbsi.re.kr (Y.-S.Y.); 4 Center for Convergent Research of Emerging Virus Infection, Korea Research Institute of Chemical Technology, Daejeon 34114, Korea; 5 Department of Physiology, College of Medicine, Chungnam National University, Daejeon 35015, Korea; harsha014@gmail.com (H.N.); cskim@cnu.ac.kr (C.-S.K.); 6 Department of Pathology, College of Medicine, Chungnam National University, Daejeon 35015, Korea; mkyeo83@gmail.com; 7 Division of Endocrinology and Metabolism, Department of Internal Medicine, College of Medicine, Chungnam National University, Daejeon 35015, Korea; 8 Brain Research Institute, College of Medicine, Chungnam National University, Daejeon 35015, Korea

**Keywords:** tauroursodeoxycholic acid, TUDCA, endoplasmic reticulum stress, ER stress, mitochondria, keloid

## Abstract

Keloids are a common form of pathologic wound healing and are characterized by an excessive production of extracellular matrix. This study examined the major contributing mechanism of human keloid pathogenesis using transcriptomic analysis. We identified the upregulation of mitochondrial oxidative stress response, protein processing in the endoplasmic reticulum, and TGF-β signaling in human keloid tissue samples compared to controls, based on ingenuity pathway and Kyoto Encyclopedia of Genes and Genomes (KEGG) analyses. Electron microscopic examinations revealed an increased number of dysmorphic mitochondria and expanded endoplasmic reticulum (ER) in human keloid tissue samples than that in controls. Western blot analysis performed using human tissues suggested noticeably higher ER stress signaling in keloids than in normal tissues. Treatment with tauroursodeoxycholic acid (TUDCA), an ER stress inhibitor, significantly decreased scar formation in rabbit models, compared to normal saline and steroid injections. In summary, our findings demonstrate the contributions of mitochondrial dysfunction and dysregulated ER stress signaling in human keloid formation and the potential of TUDCA in the treatment of keloids.

## 1. Introduction

Keloids are caused by skin injuries such as trauma, piercing, or surgery. Due to increased synthesis and reduced degradation of the extracellular matrix (ECM), excessive collagen accumulates in the dermis and the subcutaneous fat layer, resulting in the formation of excessive scar tissue in the injured area. Keloids cause esthetic issues and functional problems such as severe pain, itching, and restriction of movement at the lesion site, which affect the quality of life of the patient [1,2]. However, the etiology of excessive collagen deposition and the mechanism of symptom development remain unclear [3,4]. Genetics, [5] mechanical factors, [6] ischemia, [4] and autoimmunity [7] are implicated in keloid formation. However, its pathogenesis remains unclear, thereby inhibiting therapeutic development. Although current treatment options, including surgery, [8] silicone sheets, [9] steroid injections, [10] bleomycin injections, [11] compression, [12] laser therapy, [13] and radiation, [14] either individually or in combinations, have been used in the treatment of keloid, the recurrence rate is high. Thus, understanding the pathomechanisms of keloids and identifying novel therapeutics are crucial.

Recently, as interest in transcriptome analysis has grown, it has begun to be widely used in the field of keloid research. Through transcriptome sequencing, some studies have revealed that proteins DLEU2 and STAT3 are important in keloid regulation [15,16]. It is not easy to standardize the comparison between normal tissues and keloids because the location of keloids occurrence, their degree, and the pattern of expression for each keloid are diverse; the histological and physiological characteristics vary throughout skin layers even within a keloid lesion [17,18,19]. A study on African American patients with keloids examined the mechanism of keloid formation using bulk RNA-seq profiling of skin biopsies obtained from keloid and normal skin tissues, along with a number of transcriptome information compared to quantitative real-time PCR and immunohistochemistry [20]. The results of that study showed that immune dysregulation, particularly of Th2 and JAK3, along with Th1 and Th17/Th22 expression in keloid lesions [20]. However, they did not validate the functional study to identify the role of immune signaling in keloid development. Additionally, among the various cell types involved in keloid formation, the abnormal keloid fibroblasts (KFs) play an important role in causing cytokine imbalance [21]. The components of ECM, such as collagen, proteoglycans, elastin, and fibronectin, are also involved in keloid formation. To study the etiology and treatment of keloids, it is necessary to understand the mechanisms involved in the wound healing process, particularly the role of various cytokines as they are known to induce the movement of fibroblasts to the wound site, affect cell division and proliferation, and regulate the production of ECM components such as collagen [22].

Transforming growth factor-beta (TGF-β) is a cytokine that is implicated in wound healing and keloid formation [23]. For a substance to be effective in the treatment of keloids, it should modulate the expression of certain cytokines to indirectly intervene in the proliferation and migration of fibroblasts. The inhibition of endoplasmic reticulum (ER) stress is reportedly effective against fibroproliferative diseases such as pulmonary fibrosis [24], hepatobiliary sclerosis [25], and myocardial fibrosis [26]. However, research on the relationship between ER stress and keloids at the organelle level is lacking. In addition, the effect of ER stress on keloid scars has not been studied till date.

We investigated the major contributory regulatory signaling of human keloid pathogenesis by transcriptomic analysis of normal human skin and keloid tissues. Based on ingenuity pathway analysis (IPA) and Kyoto Encyclopedia of Genes and Genome (KEGG) analysis, we observed that the mitochondrial oxidative stress response, protein processing in ER, and TGF-β signaling were upregulated in the keloid tissues compared to that in the controls. Moreover, we validated the results through western blotting and transmission electron microscopy (TEM). In addition, we investigated the effect of targeting ER stress on keloid formation using rabbit ear scar models.

## 2. Results

### 2.1. Transcriptome Sequencing of Keloid and Normal Tissues Revealing the Link between Stress Response of Cell Organelles, including Mitochondria and ER, with Keloids

**Figure 1:**
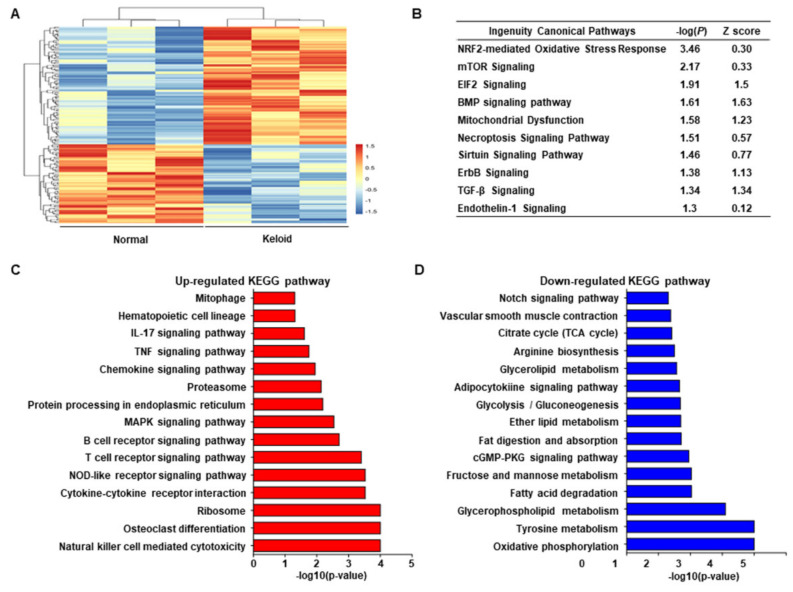
Transcriptomic analysis in keloid skin and normal skin (*n* = 3 for each group). (**A**) Repre–sentative heatmap image of differential gene expressions between keloid skin and normal skin. (**B**) Upregulated pathway in keloid using ingenuity pathway analysis (IPA). (**C**) Upregulated Kyoto Encyclopedia of Genes and Genome (KEGG) pathways in the keloid group compared to the normal group (*p* < 0.05). (**D**) Downregulated KEGG pathways in the keloid group compared to the normal group (*p* < 0.05).

To investigate the major contributory mechanism of keloids, we performed transcriptome analysis. The clinical information of the patients with keloids is shown in Table 1, and representative images of the keloids are shown in Supplementary Figure S1A. We identified significantly differentially expressed genes (DEGs) between the keloid and control groups (Figure 1A). We identified the significant canonical pathway based on unbiased analysis with the significant *p*-value and Z score using IPA. Various canonical pathways were found to be associated with keloid formation (Figure 1B, Supplementary Figure S2). Nrf2-mediated oxidative stress response, mTOR signaling, EIF2 signaling, BMP signaling, mitochondrial dysfunction, and necroptosis signaling were more enriched in keloids than in normal tissues. Additionally, KEGG analysis revealed that mitophagy, MAPK signaling pathway, and ER-related pathways (such as protein processing in the ER and ribosome pathways) were also upregulated in the keloid group (Figure 1C), whereas TCA-and glycolysis-related metabolic pathways were downregulated (Figure 1D). These results suggest a link between the stress response of cell organelles, including mitochondria and ER, with keloid pathogenesis.

### 2.2. ER Stress Signaling Was Upregulated in Keloid Tissues, Compared to Controls, in Western Blot Analysis and TEM

**Figure 2:**
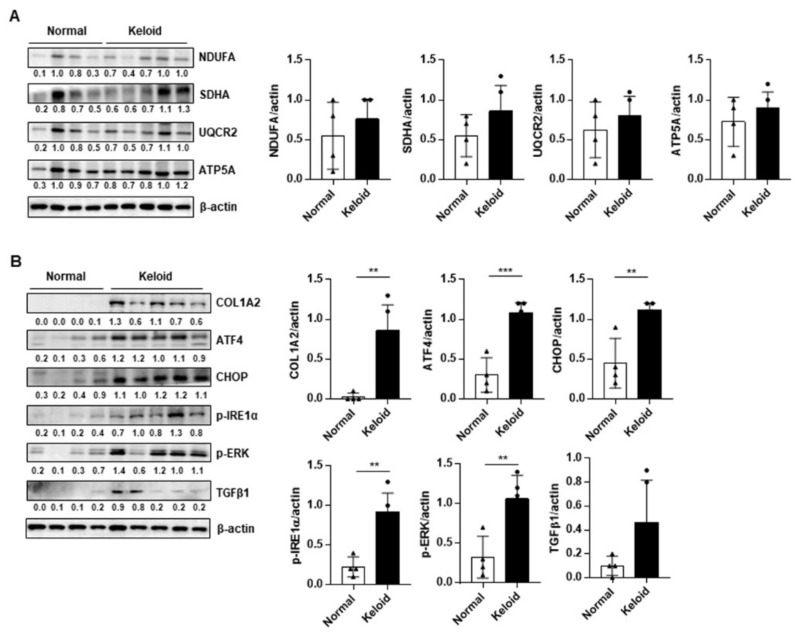
Western blot analysis of OxPhos complex subunits and endoplasmic reticulum (ER) stress signaling between normal skin tissues (*n* = 4) and keloid tissues (*n* = 5). (**A**) Representative blot and densitometry analysis of OxPhos complex subunits in normal and keloid tissues (*n* = 3 biological replicates). (**B**) Representative blot and densitometry analysis of fibrosis markers and ER stress-related genes in normal and keloid tissues (*n* = 3 biological replicates). Significance was set at *p* < 0.05. ** *p* < 0.01, and *** *p* < 0.001.

**Figure 3:**
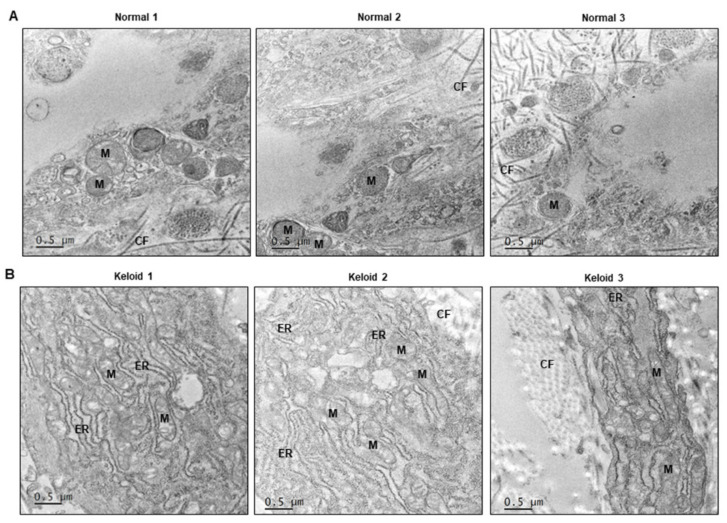
Transmission electron micrographs (EM) of normal skin (*n* = 3) and keloid tissues (*n* = 3). (**A**) Representative EM images of normal skins. (**B**) Representative EM images of keloid tissues. ER, Endoplasmic Reticulum; M, Mitochondira; CF, collagen fiber; N, Nuclues. Scale bar: 0.5 μm.

We evaluated the expression of mitochondrial OxPhos complex subunits and ER stress-related genes in four normal and five keloid tissue samples. The clinical information of the patients shown in Supplementary Figure S1B. The expression of OxPhos complex subunits, including complexes I (NDUFA), II (SDHA), III (UQCR2), and V (ATP5A), was heterogeneous in the keloid tissues, compared to that in normal tissues (Figure 2A). In contrast, collagen type 1 alpha 2 (COL1A2) and ER stress-related genes (ATF4, CHOP, ERK, and IRE1α) were significantly upregulated in the keloid tissues (Figure 2B). TGF-β expression tended to increase in keloids compared to normal tissues, however there was no significant difference (Figure 2B). To investigate the morphological change in organelles in keloid tissues compared to normal tissues, EM was performed using three normal skin tissues (Figure 3A) and three keloid tissue samples (Figure 3B). In the case of the normal skin samples, the ER appeared thin and uniformly maintained smoothly, which corresponds with general EM findings. However, in the keloid tissues, the ER appeared relatively thick and with a rough and irregular surface. Additionally, the keloid tissues contained damaged mitochondria with low density and broken cristae. These results suggest that dysmorphic mitochondria and ER stress signaling are correlated with keloid pathogenesis.

### 2.3. Tauroursodeoxycholic Acid (TUDCA), an ER Stress Inhibitor, Injection Treatment Was More Effective Compared to Steroid Injection, in Rabbit Ear Scar Models

**Figure 4:**
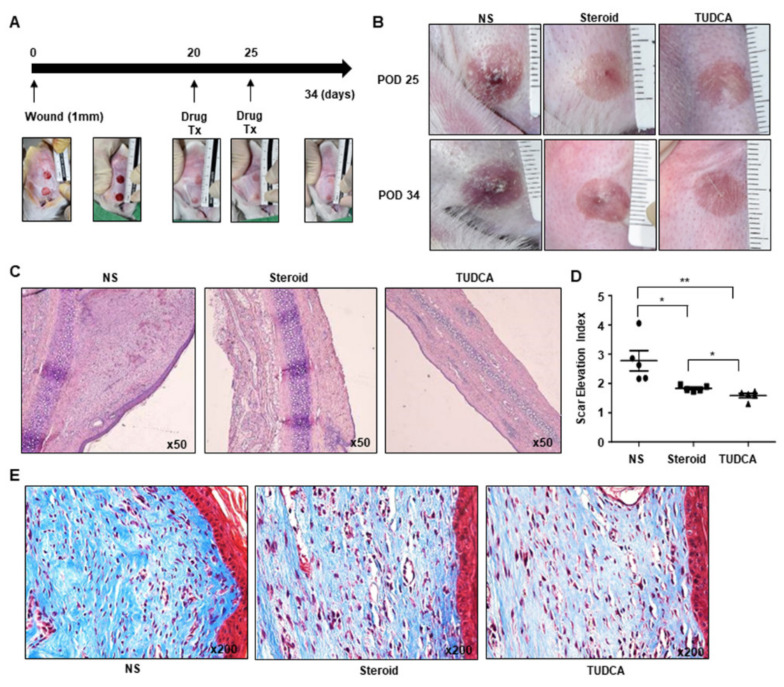
Rabbit ear scar model and the effect of TUDCA injection on keloid formation, compared to normal saline (NS) and steroid injection (*n* = 5 for each group). (**A**) Drug injection schedule and gross images. (**B**) Gross images of the scarred regions treated with NS, steroid (10 mg/mL), and TUDCA (10 mg/mL). (**C**) Hematoxylin and eosin (H&E) staining of rabbit ear scar tissues from the NS, steroid, and TUDCA groups. (**D**) Scar elevation index (SEI) calculated based on H&E staining. (**E**) Masson’s trichrome staining of rabbit ear scar tissues from the NS, steroid, and TUDCA groups. Significance was set at *p* < 0.05. * *p* < 0.05 and ** *p* < 0.01.

Next, we investigated the efficacy of TUDCA in improving scar in rabbit ear scar models (Figure 4A). Circular wounds (10 mm in diameter) were created on the ears of rabbits. After 20 days, distinctive elevated and colored scars were evident, and scarring was histologically confirmed by hematoxylin and eosin (H&E) staining. To investigate the potential of targeting ER stress in keloid treatment, we compared the effect of TUDCA with that of steroids, which are also effective in the treatment of keloids. At 20 and 25 days after wounding, normal saline (NS), steroid (10 mg/mL), or TUDCA (10 mg/mL) was injected into the scarred dermal region. The wound areas of the NS-treated group comprised palpable skin tissues resembling hypertrophic scars and appeared pale red in color. In contrast, the wound areas of the steroid- or TUDCA-treated rabbit groups exhibited better healing, exhibiting a pale pink color (Figure 4B).

To evaluate the effects of TUDCA and steroid treatment on scarring, scar tissues were subjected to H&E and Masson’s trichrome staining and observed using light microscopy. The scar tissues were harvested on day 34 after wound induction. Staining by H&E revealed the degree of scar formation. The inflammatory cells and fibroblasts were found to be irregular, and the scar tissue was thicker in the NS group compared to that observed in steroid and TUDCA groups (Figure 4C, Supplementary Figure S3). The scar elevation index (SEI) was 2.778 ± 0.346 in the control group, 1.83 ± 0.043 in the steroid group, and 1.586 ± 0.074 in the TUDCA group after treatment. Compared to the control (NS) group, both the steroid (*p* = 0.0263) and the TUDCA (*p* = 0.0098) groups exhibited statistically significant decreases in SEI. The TUDCA group also exhibited a significantly greater decrease than the steroid group (*p* = 0.0215) (Figure 4D). Staining by Masson’s trichrome revealed that the collagen fibers in the scar tissues of the NS group were generally thick and irregularly arranged. Although some irregular fiber deposition was also observed in both the steroid and TUDCA groups, the amount was lower compared to that observed in the NS group (Figure 4E).

### 2.4. Dysmorphic Mitochondria and Expanded ER Were Present in Rabbit Hypertrophic Model, Similar to That in Human Keloid Tissues, and ER Was Stabilized after Treatment with TUDCA

**Figure 5:**
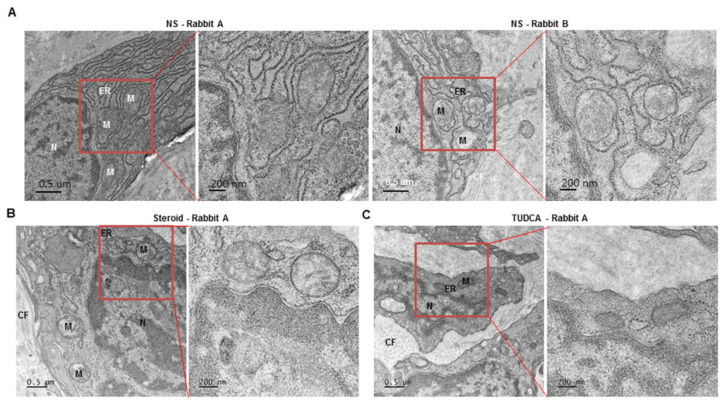
Representative images of transmission electron micrographs of rabbit ear scar models (NS group = 4 rabbits, steroid group = 3 rabbits, TUDCA group = 3 rabbits). (**A**) normal saline, (**B**) steroid (10 mg/mL), and (**C**) TUDCA (10 mg/mL). M: Mitochondria, ER: Endoplasmic Reticulum, CF: Collagen Fiber, N: Nucleus (See Supplementary Figure S3).

To observe the changes in the organelles, the rabbit scar tissue samples were observed using TEM (Figure 5, Supplementary Figure S4). In the NS group, the mitochondrial cristae were irregular in shape with a low density, while the ER had an abnormally swollen shape similar to that in the human keloid tissues (Figure 5A, Supplementary Figure S4A). In the steroid group, most ERs had a more stable and normal appearance than those in the NS group, although some ERs appeared abnormal (Figure 5B, Supplementary Figure S4B). In the TUDCA group, almost all ER exhibited normal findings, similar to those in normal human skin tissues, while the number of dysmorphic mitochondrial cristae was lower compared to the NS injection and steroid injection groups. (Figure 5C, Supplementary Figure S4C).

### 2.5. TUDCA Reduced Scarring in the Rabbit Model in Histological and Metrological Analyses through the Regulation of TGF-β1 Signaling

**Figure 6:**
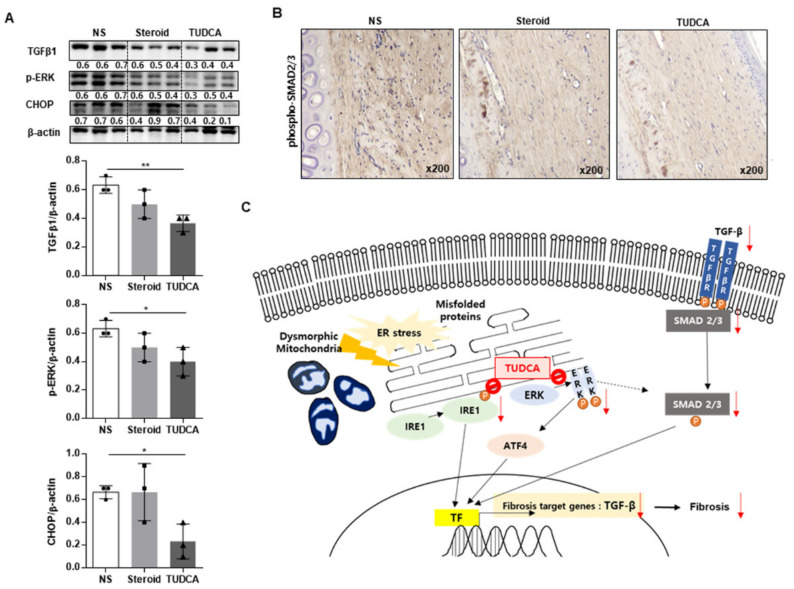
Effects of TUDCA on scar formation compared to saline or steroid, through the reduction of ER stress signaling TGF-β. (**A**) Western blot analysis of TGF-β1, phospho-ERK, CHOP, and β-actin expression in rabbit ear scars in the normal saline (NS), steroid, and TUDCA groups (*n* = 3 for each group). Densitometry of Western blot analysis in rabbit ear scars from the NS, steroid, and TUDCA groups (*n* = 3 biological replicates). (**B**) Representative images of phospho-SMAD2/3 expression in rabbit ear scars in the normal saline, steroid, and TUDCA groups using IHC (*n* = 5 for each group). (**C**) Proposed mechanism of TUDCA treatment in keloid formation. Transcription factor (TF) for TGF-β is activated through the AFT4, IRE1, and PERK pathways in keloid tissue. TUDCA inhibits the activation of IRE, ERK, and ATF4, thereby reducing TGF-β TF in the nucleus. Finally, the expression of TGF-β and fibrosis is reduced. Significance was set at *p* < 0.05. * *p* < 0.05 and ** *p* < 0.01.

To validate the mechanism of TUDCA on the regulation of TGF-β1 signaling, we investigated the expression of phospho-SMAD2/3 (p-SMAD2/3), which are the key proteins involved in the mechanism of TGF-β1 signaling. Western blot analysis revealed that TGF-β, p-ERK, and CHOP levels were significantly decreased in the TUDCA treatment group (Figure 6A). Additionally, the expression of p-SMAD2/3 was significantly reduced in the TUDCA group compared to the NS and steroid groups (Figure 6B).

## 3. Discussion

To examine the major contributing mechanism of human keloid pathogenesis, we performed transcriptomic analysis on human keloid tissues. We identified the upregulation of mitochondrial oxidative stress response, protein processing in the endoplasmic reticulum, and TGF-β signaling in human keloids compared to controls, based on IPA and KEGG analysis. Treatment with TUDCA, an ER stress inhibitor, exhibited a significant decrease in scar formation in the rabbit model compared to both NS and steroid treatments.

Keloid scars are pathological skin reactions that occur only in humans because of external stimulation [27]. These scars fail to resolve spontaneously and gradually increase in size, similar to a pseudotumor [28]. Although various treatment options are currently being used to treat keloids, the recurrence rate is high; in addition, most treatments are not limited to keloid tissue and cause atrophy and degeneration of the surrounding normal tissues [27]. Additionally, although the severity and recurrence rate of keloids differ between races, there is a limitation in that the treatment is unified. Our study is important as it suggests a new mechanism for the treatment of refractory keloids in Asian patients with moderate to severe keloids. The TGF-β1/SMAD pathway is a precise mechanism that is elevated in keloids, along with increased proliferation, differentiation, and matrix production of fibroblasts [29]. Keloid formation can be reduced by inhibiting this pathway, which is currently the most widely accepted keloid regulation mechanism [30,31]. However, as TGF-β1 signaling is also upregulated by the cytokines secreted by various immune cells surrounding the KFs. We identified the upregulation of TGF-β1 signaling along with stress response signaling of cell organelles including mitochondria and ER, using transcriptomic analysis. Our data also revealed that the inhibition of ER stress resulted in decreased keloid formation, along with the downregulation of TGF-β1 signaling.

A previous study compared keloid lesional and non-lesional skin of African American patients with keloids with normal controls, using transcriptomic analysis [20]. They identified the importance of immune dysregulation, particularly Th2 and JAK3 signaling in keloid lesions; however, the study was not sufficient to suggest a therapeutic possibility because of a lack of validation using functional studies. A recent study revealed the occurrence of lineage-specific regulatory changes in fibroblasts and vascular endothelial cells in keloids, using single-cell RNA-seq. They identified the critical regulators, including TWIST1, FOXO3, and SMAD3, involved in the fibrogenesis of KFs [32]. As TWIST1 is established to be implicated in the process of epithelial mesenchymal transition and promotion of mitochondrial dysfunction and hypoxial damage [33,34], the IPA and KEGG analysis results using bulk RNA-seq data to compare keloids and controls correspond with those of the previous studies. In addition, IPA revealed that TGF-β, mTOR signaling, endothelin, ErbB, sirtuin, and mitochondrial dysfunction differed between the keloid and the control groups, as reported in a previous study that focused on the mechanism of keloid formation [35,36,37,38]. Nrf2, EIF2, BMP, and necroptosis were also related to ER stress, both in this study and in a previous one [39,40,41,42], whereas KEGG analysis and western blotting revealed that the ER protein processing pathway was upregulated in the keloid group.

Our experimental results showed a consistent trend of increasing ER stress in keloids. The TEM images revealed that the ER in keloid tissues appeared thickened and with a rough and irregular surface compared to that in normal skin tissues, indicating elevated ER stress. By referring to the existing image analysis paper on the ultrastructure of rabbit ears, it was possible to find similar changes in human tissues as observed in the TEM images of the rabbit ear scar model [43]. Although it has been reported that the KFs contain increased numbers of autophagosomes along with mitochondrial swelling and increased vacuolization relative to NFs [44,45], ER stress has not been observed by TEM in human keloid tissues till date. Our study focused on the observation of ER stress in human keloid tissues based on TEM images. Maintaining the homeostasis of ER, which stores Ca^2+^, is important for cell survival. Furthermore, ER stress is caused by agents that perturb protein processing and folding, thereby accumulating misfolded proteins in the ER, leading to the activation of the unfolded protein response (UPR) [46]. Although there has been little research on the role of ER stress in keloids, several studies have focused on the role of ER stress in fibroproliferative diseases. For instance, in idiopathic pulmonary fibrosis, ER stress in alveolar epithelial cells has been shown to increase by external stimulation [47]. CHOP is known as the regulator for ER stress-induced apoptosis [48], as several studies have revealed that decreased CHOP and ER stress reduced the tissue fibrosis [49]. A continuous UPR activates several tyrosine kinase pathways, thereby increasing the secretion of profibrotic growth factors such as TGF-β1 and causing lung tissue fibrosis [50]. The injection of TUDCA has been shown to decrease ER stress and the expressions of TGF-β1 and Smad2/3, resulting in reduced cardiac damage [51], kidney fibrosis, and liver fibrosis [52].

Our data suggested a regulatory role of ER stress in keloid pathogenesis (Figure 6C). Since surgery, trauma, burns, and injections cause injury to the skin layer, misfolded proteins begin to accumulate, and homeostasis is broken; in addition, mitochondria undergoes dysmorphic changes if the ER stress persists. Transcription factors (TFs) for TGF-β are activated through the AFT4, IRE1, and PERK pathways. Consequently, the p-SMAD2/3 and TGF-β pathways, the major mechanisms involved in fibrosis, are exaggerated, resulting in excess ECM deposition and collagen secretion, thereby leading to fibrosis. Our analyses revealed that TUDCA acts by inhibiting the phosphorylation of ERK and CHOP, which are the ER stress pathways, thereby reducing the action of TGF-β TFs in the nucleus. Consequently, the expression of TGF-β, a fibrosis target gene, reduces and it has the potential to reduce keloid formation. However, there have been no reports on the effects of TUDCA injection on keloids. To the best of our knowledge, our study is the first to identify the therapeutic effects of TUDCA in reducing keloid scarring through TGF-β1/SMAD signaling.

With resection surgery alone, the recurrence rate of keloid is 45%–100% despite the use of silicone gel, drug injection into the lesion, cryotherapy, postoperative irradiation, or compression therapy, either individually or in combinations [53]. Among the diverse treatment methods available, steroids are the most frequently used in a clinical setting. Steroid injection reduces keloid formation; however, in some cases, it causes altered pigmentation, telangiectasia, skin atrophy, injection pain, ulceration, and cushingoid habitus [54]. Additionally, oral steroid treatment is typically not used in keloid treatment because it can cause serious complications such as infection, peptic ulcer, gastrointestinal bleeding, mental and neurological disorders, acute adrenal insufficiency, steroid withdrawal syndrome, and diabetes. A recent randomized controlled trial suggested that 5-fluorouracil (5-FU) injections may be preferable for cosmetically sensitive skin areas as it can avoid the adverse effects of steroid injections [55]. Moreover, 5-FU is established to reduce keloid scarring by blocking the synthesis of pyrimidine thymidine, a nucleoside necessary for DNA replication [56]; however, there is a lack of evidence-based recommendation for its use in the treatment of keloids as the purine synthetic pathway is also necessary for the regeneration of normal skin cells.

TUDCA is the taurine conjugate form of ursodeoxycholic acid, a hydrophilic bile acid approved by the Food and Drug Administration for the treatment of cholestasis [57]. It prevents apoptosis by regulating the mitochondrial pathway upstream of cell death, thereby reducing oxygen radical production, ER stress, and stabilizing the UPR [58]. Consequently, TUDCA has also been used to target stress response and inflammation in many in vitro and in vivo models of various diseases [58]. Recent studies have suggested that TUDCA can act as an epigenetic modulator and therapeutic agent in certain types of cancer, neurodegenerative diseases, metabolic diseases, renal diseases, and retinal disorders [59,60]. Our results are the first to indicate the potential of TUDCA in treating keloid scars. Interestingly, TUDCA-induced inhibition of scar formation was greater in the KFs than in NFs, along with upregulated ER stress in human keloids. These results suggest the potential of TUDCA to reveal the selective effects in keloid tissues compared to normal skin tissues. Although further clinical trials are required to evaluate these effects and complications, our data suggest that TUDCA may be a possible treatment option for refractory keloids.

This study, however, has some limitations. First, there was some difficulty in collecting normal skin tissue. As the sample location for each group was different, the skin characteristics of the sample sites are reflected in the transcriptome analysis, making the comparative analysis between pure keloid and normal skin more challenging [61]. Second, in the comparison of drug treatment effects on scars, this study used the thickness of scar tissue on stained slides as a parameter. However, the evaluation of color, which is one of the scar factors, was not made. In future research, it would be informative to compare thickness and color together.

## 4. Materials and Methods

### 4.1. Patient Population

This study was approved by the Research Ethics Board of the Chungnam National University Hospital, South Korea (IRB no. 2018-12-050), and informed consent was obtained from all patients.

### 4.2. RNA-Seq

Full-thickness skin was collected en bloc as 1 cm × 0.5 cm pieces from three patients with keloids and three individuals with normal skin (control group). In order to compare the superficial dermis levels, de-epithelialization was performed. At the time of surgery, 3.5x magnification loupes were worn by the professionals. The test subjects had no specific underlying diseases such as diabetes or hypertension. RNA was extracted from the keloid and normal tissues using TRIzol reagent (Invitrogen, Carlsbad, CA, USA). The quality of the extracted RNA was evaluated using an Agilent 2100 Bioanalyzer RNA Nano Chip (Agilent Technologies Inc., Santa Clara, CA, USA). The extracted RNA was used to construct RNA libraries using the TruSeq Stranded mRNA Sample Prep kit v2 (Illumina, San Diego, CA, USA), according to the manufacturer’s protocol. The quality was analyzed using an Agilent 2100 Bioanalyzer and Agilent DNA 1000 kit. Samples were sequenced using an Illumina HiSeq2500, which yielded an average of 38 million paired-end 100 nucleotide reads.

### 4.3. Transcriptomics Analysis

The reads were aligned to the UCSC Homo sapiens reference genome (GRCh37/hg19) using TopHat2 v2.1.5 (https://ccb.jhu.edu/software/tophat/index.shtml, accessed on 6 January 2019). The default TopHat parameter options were used. To analyze the differentially expressed gene (DEG) profiles between the compared groups (Control vs. Keloid), the Tuxedo protocol was used. The aligned reads were processed via Cufflinks v2.2.1 (https://github.com/cole-trapnell-lab/cufflinks, accessed on 6 January 2019), which is based on the fragments per kilobase of exon model per million reads mapped (FPKM). Unbiased, normalized RNA-seq fragment counts were used to analyze the relative transcript levels. Gene transfer format (GTF) files were generated to quantitatively compare the transcript levels in each sample to those in a reference GTF file. Next, we used Cuffdiff to calculate the differences in the FPKMs between each group. Heat maps were performed with PermutMatrix Version 1.9.3 (http://www.lirmm.fr/~caraux/PermutMatrix/, accessed on 6 January 2019).

### 4.4. Western Blot

Tissues were homogenized using TissueLyzer II (Qiagen, Hilden, Germany). Cells and tissues were lysed using the radioimmunoprecipitation assay (RIPA) buffer (30 mM Tris [pH 7.5], 150 mM sodium chloride, 1 mM sodium phenylmethylsulfonyl fluoride, 1 mM sodium orthovanadate, 1% Nonidet P-40, 10% glycerol, and phosphatase and protease inhibitors). Western blot analyses were performed with 40 μg of protein, using commercially available antibodies. Anti-CHOP, anti-pERK, anti-ERK, anti-IRE1α, anti-pIRE1α, anti-pSMAD2/3, and anti-β-actin antibodies were obtained from Cell Signaling Technology (Beverly, MA, USA). Anti-NDUFA, anti-SDHA, anti-UQCR2, and anti-ATP5A antibodies were purchased from Abcam (Cambridge, UK). Anti-COL1A2, anti-COL1A1, anti-ATF4, anti-TGF-β1, and anti-IL-1 antibodies were obtained from Santa Cruz Biotechnology (Santa Cruz, CA, USA). Peroxidase-conjugated secondary antibodies were purchased from Santa Cruz Biotechnology. Immunoreactive bands were visualized using enhanced chemiluminescence (ECL; Bio-Rad, Hercules, CA, USA). Images were scanned using the Odyssey imaging system and quantified using Image Studio Digits (LI-COR Biosciences, Lincoln, NE, USA).

### 4.5. TEM

Keloid tissue samples from patients or rabbits were fixed with 1% glutaraldehyde at 4 °C and then washed with 0.1 M phosphate buffer (pH 7.4) at 4 °C. The washed samples were post-fixed with 1% osmium tetroxide (Electron Microscopy Sciences, EMS) in 0.1 M phosphate buffer solution for 2 h at 4 °C, dehydrated using ethanol solutions (50%, 70%, 75%, 90%, 95%, and 100%), and placed in propylene oxide. The dehydrated samples were embedded progressively in 2:1, 1:1, and 1:2 mixtures of propylene oxide and EMbed-812 resins (Electron Microscopy Sciences, EMS) and polymerized at 70 °C for 24 h. The tissues were sectioned at 80 nm using a Leica Ultramicrotome (Leica, Bensheim, Germany) with diamond knives and mounted on 200 mesh copper grids. Finally, the sectioned samples were post-stained with 2% uranyl acetate and 1% lead citrate, following which the specimens were observed using a Tecnai G2 Spirit Twin TEM (FEI Company, Hillsboro, OR, USA) at 120 kV.

### 4.6. Rabbit Ear Hypertrophic Scar Model

This animal study was approved by the Chungnam National University Hospital Preclinical Laboratory Center (CNUH-019-A0079). Twelve-month-old adult female New Zealand white rabbits weighing approximately 4 kg each were purchased from Damul Science (Daejeon, South Korea). All rabbits were individually maintained in cages and provided humane care according to institutional guidelines. The experimental procedures were approved by the Institutional Review Board of the Chungnam National University Hospital. Rabbits were anesthetized using a mixture of ketamine (60 mg/kg) and xylazine (5 mg/kg), following which the ventral side of the ear was shaved and disinfected with povidone iodine. Afterward, two circular wounds were created on the ventral surface of each ear using a 10-mm biopsy punch. The skin and subcutaneous tissue layers were completely excised, and care was taken to expose the intact cartilage layer. Subsequently, the wound was covered with adhesive polyurethane dressing (Tegaderm^®^; 3M, Minneapolis, MN, USA). No antibiotics were administered. Once a day, the wound area was disinfected with normal saline (NS) and observed, and the adhesive polyurethane dressing was changed. After wound formation, a simple dressing was maintained for 20 days to form thick scar tissue. At 20 and 25 days after wounding, NS, 0.1 mL of steroid (10 mg/mL), or TUDCA (10 mg/mL) was administered to the dermal region of the rabbit ear scar using a 30-gauge syringe. Finally, 34 days after wounding, the rabbits were euthanized using CO_2_, and scar tissue was harvested en bloc.

### 4.7. Histological Analysis

Tissue samples were fixed in 10% neutral-buffered formalin for 16 h at room temperature. The samples were dehydrated, incubated in xylene, and embedded in paraffin. The paraffin-embedded tissue samples were then sectioned using a microtome, and the sections were deparaffinized, hydrated, washed, and stained using H&E and Masson’s trichrome. The slides were visualized using an Olympus BX51 microscope (Olympus, Tokyo, Japan). The H&E-stained scar tissue samples were evaluated using SEI, which provides the ratio of the total scar area to the normal underlying dermis area. An SEI of 1 indicates that the wound healed without the formation of hypertrophic dermis, whereas an SEI larger than 1 indicated hypertrophic scar formation. The SEI was calculated using ImageJ software (NIH, Bethesda, MD, USA). Four-micrometer-thick sections of paraffin-embedded tissue blocks were incubated at 56 °C for 3 h before immunohistochemistry analysis. Specimens were stained using the Vectastain ABC HRP kit (Vector Laboratories, Inc., Burlingame, CA, USA). Antigen retrieval was performed by microwaving in citrate buffer for 10 min. Endogenous peroxidase activity was inactivated by incubation in 3% hydrogen peroxide for 10 min. Non-specific binding sites were blocked by incubation in 10% normal goat serum in phosphate-buffered saline (PBS). Tissue section slides were incubated for 1 h at room temperature with primary antibodies against phospho-SMAD2/3 (1:100, Cell Signaling). The tissue sections were then counterstained with hematoxylin. Negative controls were incubated with PBS rather than the primary antibody, and no positive staining was observed. The tissue slides were analyzed using an Olympus BX51 microscope. Two certified pathologists performed the microscopic analyses and were unaware of the identity of the samples or the corresponding clinicopathologic data.

### 4.8. Statistical Analysis

Results are expressed as the mean ± SD. In western blot analysis performed using human tissues, we used distinct tissues from 4 control donors and 5 keloid donors, and compared the mean expression between the two groups. To evaluate the effects of TUDCA or steroid injections in rabbit scar models, we used 5 rabbits in each group. Technical replications were performed with at least three independent experiments in western blot analysis and 2 wounds per rabbit ear. Results were evaluated using one-way analysis of variance or Student’s two-tailed *t-*tests. Statistical significance was set at *p* < 0.05. Statistical analysis was performed using the SPSS software (version 22.0; IBM Corp., Armonk, NY, USA).

## 5. Conclusions

We found that mitochondrial oxidative stress response, protein processing in endoplasmic reticulum, and TGF-β signaling were implicated in human keloids compared controls using transcriptomics analysis. We also identified the role of ER stress signaling in keloid pathogenesis, based on the effects of TUDCA treatment on keloid formation in in vivo model. In conclusion, the present results demonstrate the contributions of mitochondrial dysfunction and dysregulated ER stress signaling in human keloid formation and the potential of TUDCA in the treatment of keloids.

## Figures and Tables

**Table 1:** Baseline characteristics of the patients who provided samples for RNA-Seq.

Patient	Group	Race	Age	Site	Etiology	Lesion Duration	Lesion Size	Prior Treatments	Keloid Family History
1	Control	East Asian	43/F	Abdomen	Normal	Normal	Normal	Normal	Normal
2	Control	East Asian	28/F	Chest	Normal	Normal	Normal	Normal	Normal
3	Control	East Asian	36/F	Flank	Normal	Normal	Normal	Normal	Normal
4	Keloid	East Asian	31/F	Ear	Piercing	7 years	4x2cm	Excision	Keloid
5	Keloid	East Asian	33/F	Ear	Piercing	8 years	3x1cm	Excision	Normal
6	Keloid	East Asian	23/F	Ear	Piercing	5 years	2x2cm	None	Normal

## Data Availability

Publicly available datasets were analyzed in this study. This data can be found here: https://www.ncbi.nlm.nih.gov/sra/PRJNA764368.

## Supplementary Materials

Supplementary Materials are available online at https://www.mdpi.com/article/10.3390/ijms221910765/s1.
