# Supplementary figures and images for: Tauroursodeoxycholic Acid Decreases Keloid Formation by Reducing Endoplasmic Reticulum Stress as Implicated in the Pathogenesis of Keloid
Source: Int J Mol Sci. 2021 Oct 5;22(19):10765. doi: 10.3390/ijms221910765 (PMC8509846; doi:10.3390/ijms221910765)

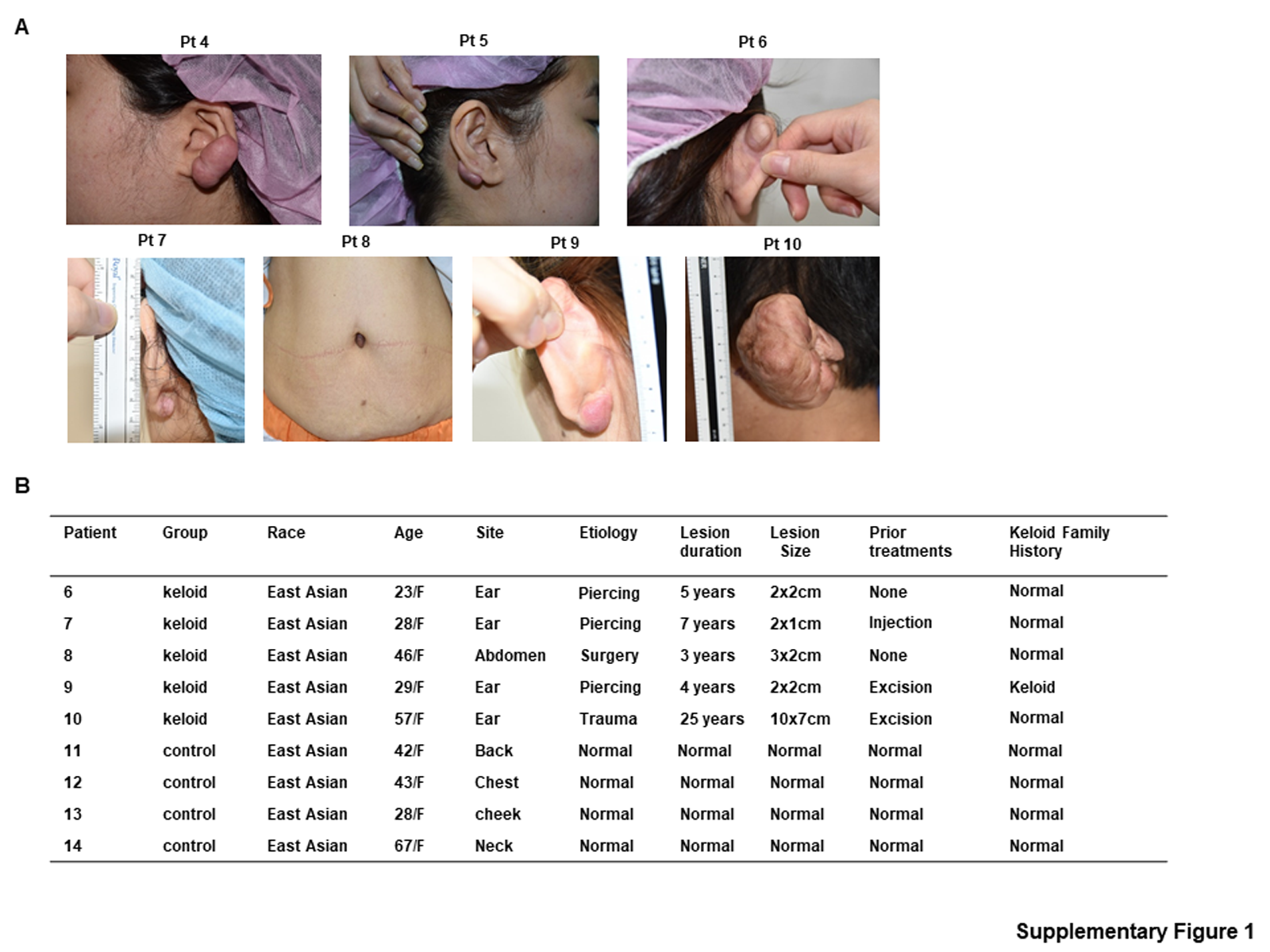

Supplement: Supplementary file 1 [file ijms-22-10765-s001.zip › Supplementary Figures/Supplement figure 1.tif]

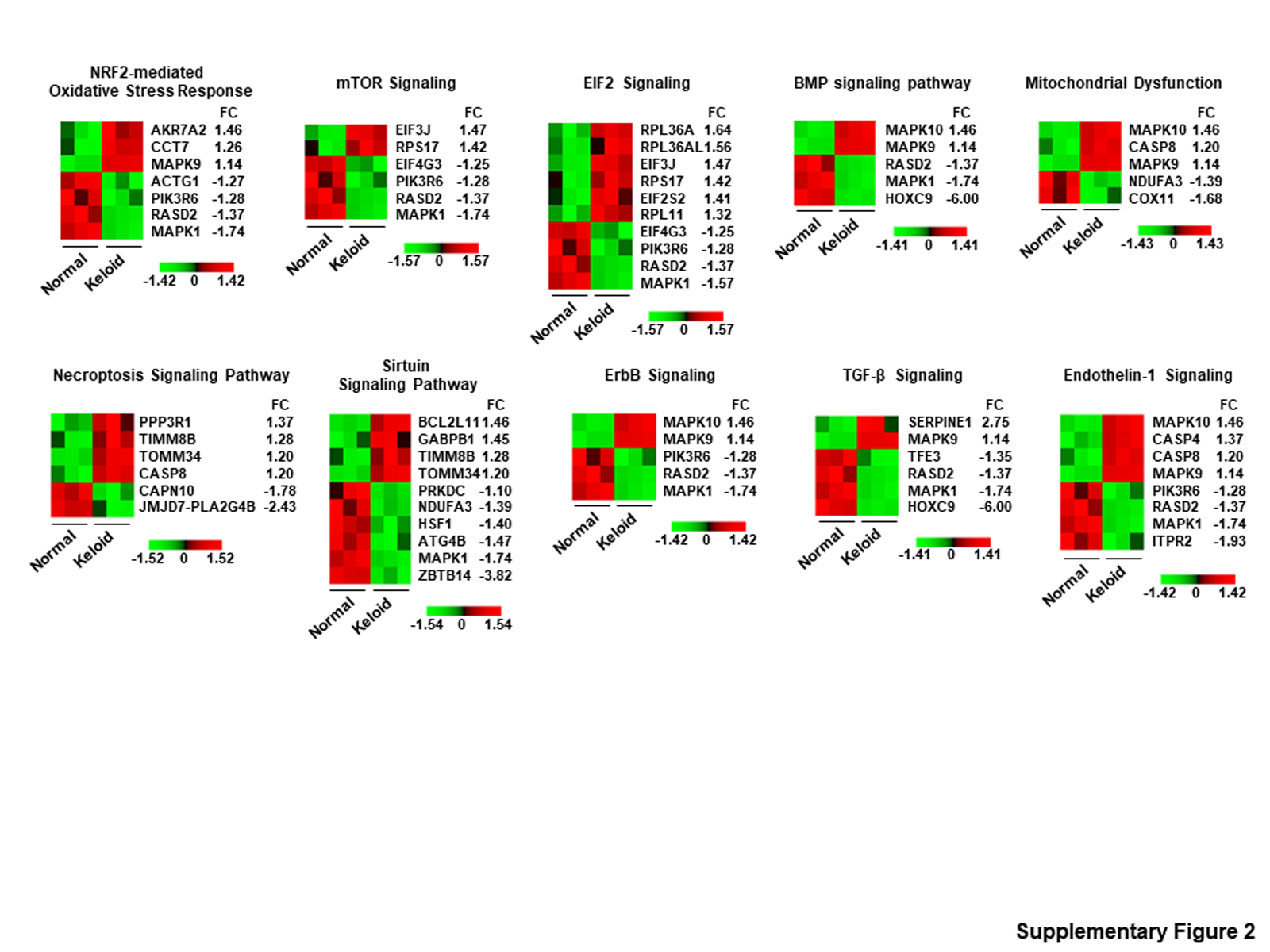

Supplement: Supplementary file 1 [file ijms-22-10765-s001.zip › Supplementary Figures/Supplement figure 2.tif]

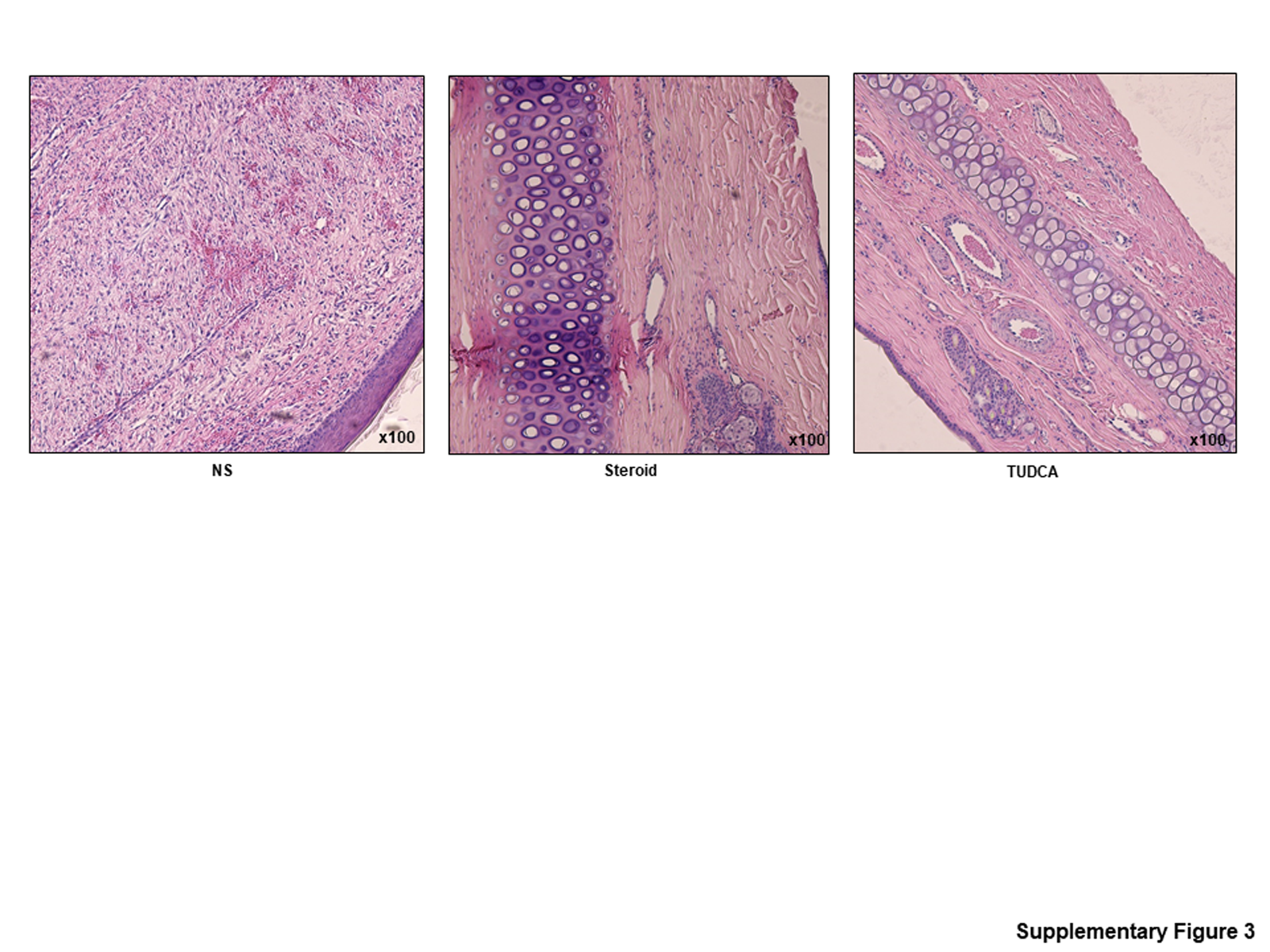

Supplement: Supplementary file 1 [file ijms-22-10765-s001.zip › Supplementary Figures/Supplement figure 3.tif]

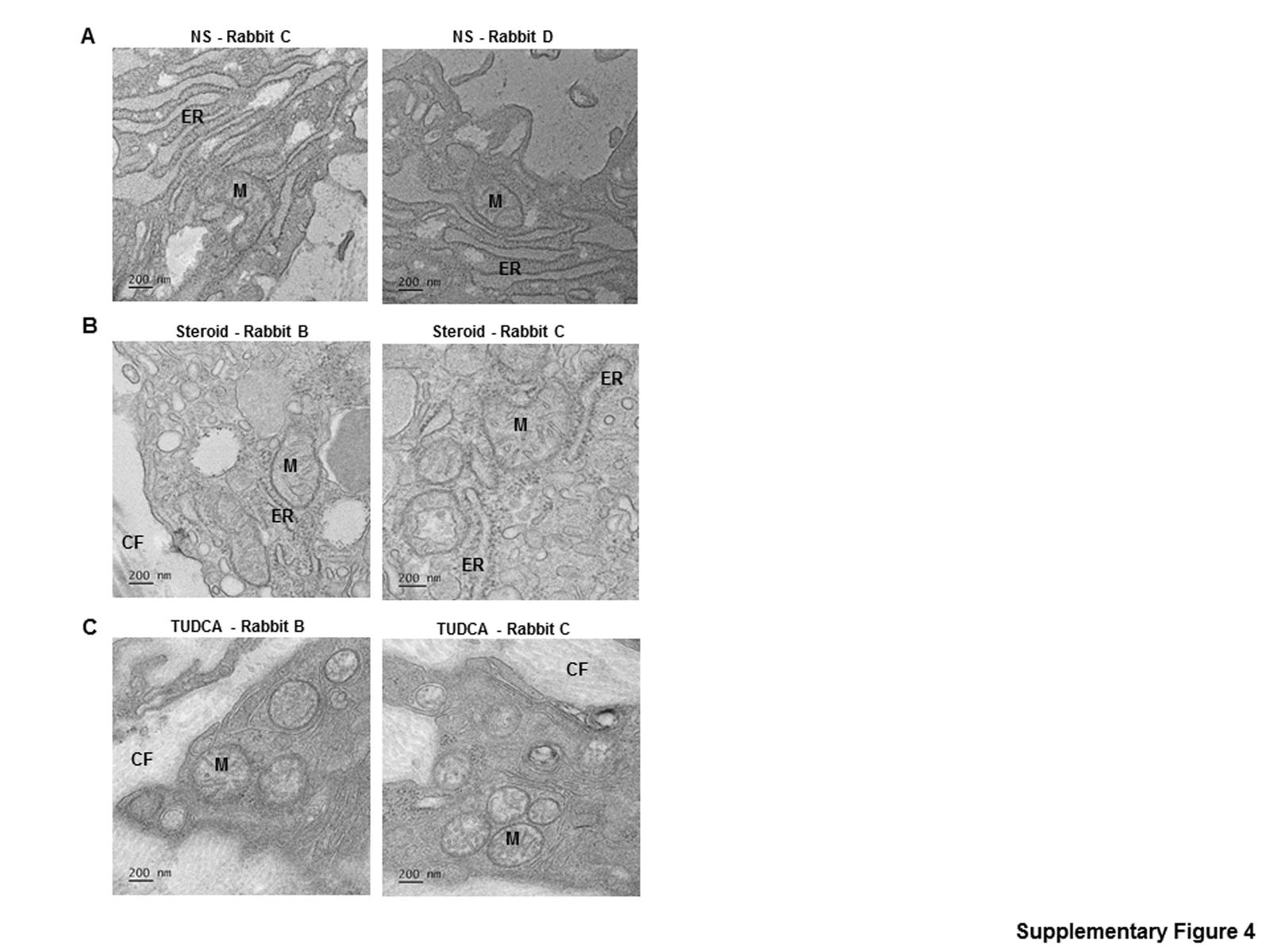

Supplement: Supplementary file 1 [file ijms-22-10765-s001.zip › Supplementary Figures/Supplement figure 4.tif]
